# Mediating Effect of Sports Safety Awareness between Sports Activity Habits and the Intention to Complete Safety Education among Korean Adolescents

**DOI:** 10.3390/healthcare11131891

**Published:** 2023-06-30

**Authors:** Ki-Hee Jo, Seung-Man Lee, Wi-Young So, Eui-Jae Lee

**Affiliations:** 1 Center for Textbook Authorization, Department of Curriculum and Textbook, Korea Institute for Curriculum and Evaluation, Jincheon-gun 27873, Republic of Korea; kiheejo@kice.re.kr; 2 Department of Sports Science, Hankyong National University, Anseong-si 17579, Republic of Korea; lsm14pe@hknu.ac.kr; 3 Sport Medicine Major, College of Humanities and Arts, Korea National University of Transportation, Chungju-si 27469, Republic of Korea; 4 Department of Physical Education, Graduate School of Education, Sogang University, Seoul 04107, Republic of Korea

**Keywords:** adolescent, intention to complete safety education, mediating effect, sports activity habits, sports safety awareness, structural equation model

## Abstract

Background: As the importance of safety during sports activities continues to gain emphasis socially, the interest in creating a culture of safety and safety education to support this is also increasing. However, no study has examined the willingness of adolescents to complete safety education voluntarily. To identify methods of building a culture of sports safety among adolescents, this study investigated the structural relationship among three related variables: sports activity habits, intention to complete safety education, and sports safety awareness of Korean adolescents. Methods: Data on 3928 adolescents aged 13–18 years old from the 2019 Sports Safety Accident Survey conducted by the Korea Sports Safety Foundation were analyzed. This encompassed frequency analysis, scale reliability, validity verification, descriptive statistics analysis, path analysis, and mediating effect verification. Results: The results indicated that sports activity habits among these adolescents had a positive effect on their sports safety awareness and on their intention to complete safety education; further, their sports safety awareness had a positive effect on their intention to complete safety education. The results also showed that sports safety awareness had a partial mediating effect between sports activity habits and intention to complete safety education. The willingness of adolescents to voluntarily complete safety education is particularly important, as adolescence is a critical period when lifelong safety habits can be formed. Conclusions: Based on the results, discussions on creating safe sports activity habits for adolescents and continuous education on sports safety awareness are needed. Ultimately, we need to improve sports safety awareness by paying attention to the development and implementation of sports safety education programs for adolescents as a national policy and, through this, increase their willingness to complete safety education.

## 1. Introduction

With an increased emphasis on safe participation in sports, interest in creating a culture of safety has grown in society [1,2]. Accordingly, safety education for adolescents, who often participate in a multitude of different sports both inside and outside school, needs special attention, as this is a critical period when safe lifestyles are formed. Safe participation cannot be overemphasized; therefore, the willingness to voluntarily complete safety training is increasingly important [3,4,5]. Recently, during the COVID-19 epidemic, the interest in training and sports in Korea increased; YouTube and TV programs (e.g., soccer-based “Come Together to Kick,” futsal-based “Goal Hitters,” tennis-based “Tomorrow’s Winning Shot”) that offer such participation gained popularity. As interest in sports has increased, the number of participants in sports has also increased rapidly. Concurrently, various accidents have occurred during sports activities.

Sports participation can be divided into professional elite athletes (who are paid to participate in the sport) and those who participate for enjoyment. Many injuries occur among elite youth athletes, but they also occur frequently during school physical education for middle and high school students and ongoing life sports [1,2,3]. Emery and Tyreman [2] investigated the injury rate of adolescents participating in sports in Calgary, Canada, and reported that approximately 61 out of 100 adolescents experienced injuries. Thus, greater measures to prevent adolescent accidents when participating in sports are required.

For adolescents to enjoy sports safely over a long period of their lives, a safe sports lifestyle is necessary. Most injuries can be prevented depending on the habits of those participating in the sport, while secondary injuries can be prevented with prompt post-injury first aid. However, developing safe habits in sports activities requires participation in sports as a basic premise. Studies on sports participation have shown that it is not necessarily easy for everyone to participate in every sport, even if they want to [6]. That is, participation may be difficult based on the economic and social environment. Nevertheless, if one is able to participate in sports, safe activity habits can prevent injuries regardless of sports activity frequency and lead to greater overall participation [7].

Several studies on sports safety have focused on accidents that occur during sporting events [8,9]. Some scholars have reported on the need for more studies on the prevention of accidents, rather than injuries, in sports [4,10,11,12]. Other researchers have focused on methods for preventing injuries in various sports, while some have looked at the development of sports safety education programs [11,13,14] and the use of digital devices to improve sports safety [15,16]. As most studies on the topic are focused on preventing safety-related injuries while participating in sports, studies on accident prevention in sports situations from a new perspective are required [13].

Safety accidents may occur because of negligence or complacency on the part of the participant, but there are also cases where accidents are caused by others. Therefore, attention to the safety awareness of sports participants is needed. Lee et al. [3] compared the safety awareness of elite soccer players with that of soccer club players. In various fields of safety awareness, such as awareness of safety rules by event, awareness of how to prevent accidents in sports, awareness of how to cope with accidents in sports, and first aid tips for accidents in sports, elite soccer players showed higher safety awareness than that of sports club members. Thus, some have argued that safety awareness among life sports club members should be increased. Additionally, when looking at previous studies related to running, exercise habits, and safety awareness involving amateur athletes, it was found that various factors such as participation time, frequency, gender, age, injury experience, and location affect safety awareness [17,18,19].

In Korea, physical education is a compulsory element of the national school curriculum. Thus, attention needs to be given to safety education that cultivates safety awareness among adolescents. Through such education, these students can prepare for accidents, respond quickly and decisively when an injury occurs, and be responsible for the safety of others. This can then become a virtual cycle that leads to the creation of a safe sports lifestyle.

Research related to safety in sports situations is now attempting to drive legislation on safety education, with considerable support from a policy standpoint and not simply from the perspective of prevention or compliance with safety rules. In addition to the United States, various countries have indicated that there should be policies for adolescent or adult participation in sports related to safety, injury prevention programs, and preventive education [20,21,22,23]. As such, various complex studies have been conducted [5,9,11]; however, to the best of our knowledge, no study has focused on the relationship between sports activity habits, sports safety awareness, and safety education. Our study fills this gap by focusing on adolescents’ willingness to complete safety education. In one similar study, an experiment found no direct effect of fitness apps on fitness center users’ continuing intentions and sports habits [24]. Another similar study looked at the effect of physical self-concept on intrinsic motivation and sports habits [25]. However, to the best of our knowledge, no study has examined the willingness of adolescents to complete safety education voluntarily. As school education cannot fully cover all potential accidents or how to deal with them, adolescents’ willingness to complete a targeted sport safety education program is critically important.

**Figure 1:**
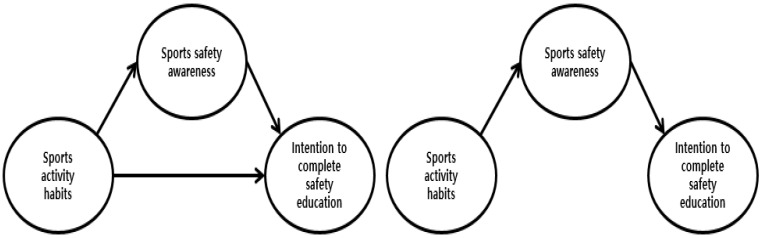
Hypothesis model and competition model.

Our study investigated the structural relationships among sports activity habits, the awareness of sports safety, and the intention to complete safety education for Korean adolescents. We also specifically investigated the mediating effect of the awareness of sports safety between sports activity habits and the intention to complete safety education. For our analysis, we developed the following hypotheses for adolescents: sports activity habits will positively affect sports safety awareness (Hypothesis 1); sports activity habits will positively affect the intention to complete safety education (Hypothesis 2); sports safety awareness will positively affect the intention to complete safety education (Hypothesis 3); and sports safety awareness will be a mediating effect between sports activity habits and the intention to complete safety education (Hypothesis 4). Specifically, sports safety awareness will partially mediate (hypothesis model) or fully mediate (competition model) between sports activity habits and the intention to complete safety education, as shown in Figure 1.

## 2. Material and Methods

### 2.1. Participants

Our participant data were obtained from the 2019 Sports Safety Accident Survey conducted by the Korea Sports Safety Foundation (KSSF). The KSSF [26] conducts a Sports Safety Accident Survey every 4 years as part of its sports safety research. The KSSF formed a population (as of June 2019) using the resident registration statistics of the Korea Ministry of Government Administration and Home Affairs, which can identify the population distribution ratio by region, gender, and age in Korea on a monthly basis to secure raw data related to sports safety. In addition, KSSF set the population of the study to Korean citizens who can express their subjective opinions. KSSF was conducted as an online panel web survey based on the online panel of an already established survey agency. Since we adopted stepwise mailing, a common web search method, that is, a random mailing method for the same multiple for each assignment, it can be said that random sampling is guaranteed without any possibility of using arbitrary criteria in the extraction process. As such, sampling was performed using the quota sampling method, which proceeds randomly within the allocation.

The survey was conducted for approximately 3 months, from 20 September 2019 to 24 December 2019. A total of 7725 participants defined as sports players for life (hereafter club sports players) and 4020 professional sports players were surveyed nationwide. Of them, 11,745 participants (7452 males and 4293 females) responded to the survey; all participants responded without fail (no missing data). In this study, among the raw data provided by KSSF, the data of 3928 adolescents aged 13–18 years old suitable for the purpose of this study were used (Table 1). The KSSF’s raw data did not include personal identification information, such as names, phone numbers, home addresses, or social security numbers; therefore, it was not necessary to go through the ethical approval process. All study procedures were approved by the KSSF and were conducted in accordance with the principles outlined in the Declaration of Helsinki.

### 2.2. Instruments

Among the KSSF questionnaire items, we looked at 21 questions to assess and measure the three variables in our research model. First, the sports safety activity habits variable comprised a total of 10 items: (1) pre-exercise warm-up; (2) checking the surrounding facilities for defects and safety before exercise; (3) performing appropriate exercise after determining the exercise level beforehand; (4) adequate intake of water before exercise; (5) drinking water during the designated break time; (6) wearing appropriate clothing and safety gear for the sport; (7) reporting pain and injuries to the coach; (8) checking environmental factors, such as weather disasters and signs before exercising; (9) being aware of first aid methods, such as cardiopulmonary resuscitation (CPR); and (10) engaging in cool-down exercises. Second, the sports safety awareness variable comprised eight items: (1) awareness of how to prevent sports accidents; (2) awareness of how to cope with sports accidents; (3) awareness of CPR; (4) awareness of wound disinfection and use of bandages; (5) awareness of coping with musculoskeletal damage; (6) awareness of coping with accidents caused by diseases; (7) awareness of coping with physical damage caused by shock; and (8) awareness of coping with temperature change accidents. Finally, the intention to complete safety education variable comprised three items: (1) necessity of sports safety education; (2) intention to complete safety education; and (3) intention to complete a specific safety education program. The questions were measured on a five-point Likert scale with 1 being “not at all” and 5 being “always”, with each score calculated independently.

### 2.3. Reliability of Instruments

To check the reliability of the model we created for our study, we used Cronbach’s α to verify the consistency of the items and confirmatory factor analysis to verify validity. Table 2 presents the reliabilities of the variables. Cronbach’s α for sports activity habits was 0.811, 0.868 for sports safety awareness and 0.685 for the intention to complete safety education; all were above the standard value of 0.6; therefore, it can be evaluated as satisfying internal consistency reliability [27].

Next, we verified the convergent, discriminant, and nomological validity of the model using confirmatory factor analysis. For the goodness-of-fit of the confirmatory factor analysis, the incremental fit index (IFI) and the comparative fit index (CFI) were verified; the absolute fit index was verified through the root mean square residual (RMR), the goodness-of-fit-index (GFI), the root mean square error of approximation (RMSEA), and χ^2^/degree of freedom (DF). The results are shown in Table 3. However, six items were removed based on the square multiple correlation (SMC) value because all indices were below standard. The GFI of the modified model was higher than its standard value. Table 3 shows whether the confirmatory factor analysis for the proposed and modified models is appropriate.

The validity of the revised model was then verified using confirmatory factor analysis, as shown in Table 4. First, to verify convergent validity, we used three methods: standardized regression coefficient, mean variance extraction, and conceptual reliability. The range of standardized regression coefficients of all variables was 0.388–0.919, and the significance of the critical ratio (CR) was 1.965 or more. In addition, the construct reliability was 0.990–0.995 and the average variance extracted (AVE) was 0.932–0.977, indicating convergent validity. Subsequently, we verified nomological validity. In this study, the relationship between constructs was predicted in the positive (+) direction, and as a significantly positive (+) relationship between latent variables actually appeared, we were able to confirm nomological validity.

To identify the discriminatory validity of the revised model, we compared the correlations between the constructs and the AVE, as shown in Table 5. The square of the correlation coefficient of “sports activity habits ↔ sports safety awareness” (most correlated) was 0.254 and was lower than the AVE of sports activity habits (0.932) and sports safety awareness (0.977). Therefore, we confirmed the discriminant validity among the variables.

### 2.4. Procedure and Statistical Analysis

As noted above, we gathered data on 3928 adolescents and conducted our analysis using SPSS and AMOS (version 18.0; IBM Co., Armonk, NY, USA). Our methodology included the following: (1) frequency analysis to confirm the general characteristics of study participants; (2) Cronbach’s α to evaluate the reliability of items in our model; (3) confirmatory factor analysis to verify the validity of the model and verification of concentration, discrimination, and nomological validity; (4) descriptive statistical analysis to confirm the participants’ awareness of each variable; (5) verification of the goodness-of-fit of the model to confirm the structural relationship of each variable and path analysis; and (6) bootstrapping to verify the mediating effect of sports safety awareness between adolescent sports habits and intention to complete safety education.

Shrout and Bolger [28] used 10,000 bootstrapping samples generated by a random sampling of the original data for parameter estimation because it is difficult to guarantee that the mediating effect follows stationarity. For this, we followed the suggestion of setting a 95% confidence interval (CI). In addition, we investigated the indirect effect of sports activity habits via sports safety awareness on intention to complete a safety education program. Significance was set at *p* < 0.05.

## 3. Results

### 3.1. Descriptive Statistical Analysis

All factors and sub-factors were analyzed to confirm the descriptive statistics on three variables: sports activity habits, sports safety awareness, and the intention to complete safety education. The results are presented in Table 6. The mean was between 3.19 and 4.49, and the standard deviation was between 0.672 and 1.007. In terms of skewness and kurtosis, if skewness <±3.00 and kurtosis <±10.00, the criteria that violate univariate normality, the normal distribution can be evaluated as satisfying the conditions [29,30]. In our study, the absolute value of skewness was between 0.084 and 1.348, and the absolute value of kurtosis between 0.085 and 2.272, thereby satisfying the condition of stationarity of the structural equation.

### 3.2. Path Analysis

As noted, our structural model consisted of three latent variables: sports activity habits, the awareness of sports safety, and the intention to complete safety education. In the revised model, these were measured based on 15 items: (1) pre-exercise warm-up; (2) checking the surrounding facilities for defects and safety before exercise; (3) performing the appropriate exercise after determining the exercise level beforehand; (4) drinking an adequate intake of water before exercise; (5) wearing appropriate clothing and safety gear for the sport; (6) reporting pain and injuries to the coach; (7) checking environmental factors such as weather disasters and signs before exercising; (8) engaging in cool-down exercises; (9) being aware of how to prevent sports safety accidents; (10) being aware of how to cope with safety accidents in sports; (11) being aware of CPR; (12) being aware of coping with musculoskeletal damage; (13) understanding the necessity of sports safety education; (14) intending to complete safety education; and (15) intending to enroll in a sports safety program.

We followed a structural equation analysis procedure, as suggested by Anderson and Gerbing [31]. Thus, we verified our measurement model using a two-step approach and then performed path analysis. As a result of the path analysis and checking the GFI of the entire model to verify the direct and indirect effects, our model showed generally acceptable GFI, as shown in Table 7.

For hypothesis testing, we analyzed the causal relationship between each variable in the model. Our results support our first three hypotheses, as shown in Table 8. Specifically, for Hypothesis 1, the path coefficient of the hypothesis that “sports activity habits will have a positive effect on sports safety awareness” was 0.602 (t = 20.949), confirming the hypothesis; for Hypothesis 2, the path coefficient of the hypothesis that “sports activity habits will have a positive effect on the intention to complete safety education” was 0.360 (t = 12.252), confirming the hypothesis; finally, for Hypothesis 3, the path coefficient of the hypothesis that “sports safety awareness will have a positive effect on the intention to complete safety education” was 0.145 (t = 5.873), confirming the hypothesis.

### 3.3. Mediating Effect of Sports Safety Awareness between Sports Activity Habits and Intention to Complete Safety Education

We conducted the following analyses to test the mediating effect of sports safety awareness in the relationship between adolescents’ sports activity habits and their intention to complete safety education (Hypothesis 4). We also verified the model that explained the structural relationship of each variable. First, there was no partial mediation model (hypothesized model); thus, there were no direct paths between sports activity habits and the intention to complete safety education that directly affected the intention to complete safety education or the recognition of sports safety. However, by analyzing and comparing the fitness of the fully mediated model (competition model) in which sports activity habits affected the intention to complete safety education due to the awareness of sports safety, we searched for the simplest optimal model that best explained the data. In addition, as shown in Table 9, we also identified a fit index to compare the research and competitive models.

As the fully mediated model was embedded in the partially mediated model, we performed the χ^2^ difference test. The difference value for the goodness-of-fit of the two models to be statistically significant at 0.05 in the χ^2^ difference test is 5.99 DF. Through our verification, the value of χ^2^ difference between the two models was 189.148 and the difference in DF was 1.00, showing a statistically significant difference at the 0.05 level. If the result of the χ^2^ difference test was statistically significant, the partial arbitration model was selected; otherwise, the full mediation model was selected [32]. The full mediation model set as a hypothesis model in this study means that independent variables do not directly affect dependent variables but affect them through mediated variables. Therefore, in our study, the partial mediation model, which is a hypothetical model, was selected as the final model. That is, the direct effect of sports activity habits on the intention to complete safety education and the indirect effect of sports activity habits on the intention to complete safety education through sports safety awareness were significant.

The bootstrapping method proposed by Shrout and Bolger [28] was used to verify the indirect effect of sports safety awareness on the relationship between sports activity habits and the intention to complete safety education. This method can evaluate the standard error of the indirect effect that can accompany the existing mediating effect test with a confidence interval; if the interval does not include 0.00, the indirect effect is regarded as statistically significant. The indirect effect (β = 0.177, 95% bias-corrected CI = 0.144–0.212) of sports activity habits on sports safety awareness and the intention to complete safety education was statistically significant, as shown in Table 10. That is, the higher the sports activity habits, the higher the awareness of sports safety and, ultimately, the higher the intention to complete safety education.

## 4. Discussion

This study aimed to analyze the structural relationship among sports activity habits, sports safety awareness, and the intention to complete safety education in adolescents. As such, this study was able to verify the mediating effect of sports safety awareness between sports activity habits and the intention to complete safety education. These results can now be compared with those of previous related studies.

First, as confirmed in Hypothesis 1, here, it was found that sports activity habits had a positive effect on sports safety awareness. Here, sports safety awareness refers to the degree of knowledge about first aid measures in cases of various injuries and accidents, as well as overall sports prevention and coping methods. It was confirmed that behaving safely in sports activities had a positive impact on the cognitive aspects of knowing how to play sports safely. This is consistent with the results of previous studies showing that sports participation rates and habits or behaviors while participating in sports directly affect safety accidents and injury prevention in sports [2,33,34]. In addition, Neal and Griffin [9] found a high correlation between creating a safety culture and a safe atmosphere, depending on how sports behavior is practiced. In this way, creating active habits among adolescents to engage in sports safely can contribute not only to the development of adolescent awareness of safety in sports, but also to the creation of a safe culture. It can be concluded that crucial efforts and strategies are needed to develop safe sports activity habits and prevent accidents in sports [34]. Adolescence is an important period directly related to activity habits in adulthood. Sports safety promotional materials that can be easily accessed should include safety education, manuals/guidelines, checklists, and posters [8]. Therefore, for adolescents participating in sports, manuals, guidelines, and lists of behavioral regulations should be developed, along with educational materials that can lead to safe activity habits, divided into general and individual sports. By using such information, students can assess and manage their behavior and develop correct habits.

Second, as stated in Hypothesis 2, sports activity habits have a positive effect on the intention to complete safety education. The intention to complete safety education refers to the willingness of the sport’s participants to voluntarily learn about accident prevention and coping methods. Several previous studies have developed safety education programs for adolescent participation in sports, and various studies have suggested directions for improving safety education [11,13,35]. Regardless of how good safety educational content or programs are, if the sport’s participants do not intend to voluntarily participate in the education, the programs will prove to be ineffective. This study focused on the willingness to complete safety education, which is important but often overlooked. Here it was investigated whether safety education should be completed according to how participants participate in sports and their activity habits. These results concluded that it is important to devise methods and strategies for developing safe activity habits while paying attention to the willingness of the participants to complete safety education, which has been overlooked thus far.

Third, as stated in Hypothesis 3, sports safety awareness was found to positively affect the intention to complete safety education. Just as sports activity habits had a positive effect on safety awareness and intention to complete safety education, safety awareness also had a positive effect on the intention to complete safety education. This is similar to the results of Kim and Lee [36], who found that safety awareness and safety education had a close relationship. Thus, we need to begin by raising safety awareness through continuous education on sports safety, paying attention to the research result that the higher the awareness of safety in sports participation, the higher the willingness to complete safety education. Examining various previous studies related to safety awareness, studies have reported that safety awareness should be continuously promoted and practiced, and safety education should be implemented in sports situations as a policy [4,13,37,38]. In addition, as the number of emergency cases due to cardiac arrest has increased, for example, in the English Premier League, safety training should be required to provide first aid during games when an emergency occurs [39]. Based on these results, to improve sports safety awareness, efforts need to be made to enhance the actual sports site as a place for practical sports safety education.

Finally, the analysis of Hypothesis 4 revealed that sports safety awareness partially mediated the relationship between sports activity habits and intention to complete safety education. These results indicate that sports activity habits directly affected the adolescent’s intention to complete safety education, while sports safety awareness had a mediating effect on the intention to complete safety education. Human factors that influence adolescent awareness of sports safety include coaches, physical education teachers, and parents [4,40,41]. Safety education for elite players and school physical education students should include similar considerations based on sports safety-related knowledge gathered through trial and error, as the exercise systems created by physical education teachers and sports team leaders are not autonomous exercise environments. The leaders in both cases should emphasize the importance of safety education and pay attention to improving safe participation habits and awareness of sports safety. If sports safety education is conducted intentionally and systematically, adolescent sports safety can be further improved. This investigation of these three variables is a novel approach to this topic not addressed in previous studies. The three variables of sports activity habits, safety awareness, and intention to complete safety education are closely related and form a virtually connected cycle of influence. Based on these results, with proper education, adolescents can participate in and enjoy sports without injury. A safe sports environment can be designed so that direct safety education can be conducted in the field based on the experience and the know-how of the educators and team leaders involved.

However, there are some limitations to our study that warrant further discussion. First, although we considered basic sports activity habits, such as exercise duration, participation time, and number of participants, we excluded personal characteristics, such as safety education experience or injury experience. Thus, variations in the results may have occurred depending on the individual characteristics of the participants. Second, the study used a quantitative survey. Therefore, there was a lack of qualitative stories about the process beyond the relationship among adolescent sports activity habits, sports safety awareness, and the intention to complete safety education. Future research could follow up with qualitative research that confirms this relationship in greater depth and derives implications through direct conversations with the participants. Third, in the sports science field, it is important to analyze issues between elite athletes and club sports players separately, by sport type (e.g., track and field, soccer, rugby), by intensity and frequency of sports participation, and by the presence or absence of the past experiences of sports-related injuries. However, this study did not investigate each of these issues; therefore, in the future, further studies are necessary. Fourth, we looked only at adolescents in Korea, where there is an emphasis on safety in physical education in the national curriculum. In the future, similar studies targeting adolescents from various countries should be considered, and comparative studies according to the economic and social levels of each country should be conducted. In addition, studies exploring the effects of developing and operating sports safety education programs in each country could be investigated. Fifth, this study drew meaningful results on sports safety for adolescents participating in sports but did not consider various characteristics of sporting events. In future studies, it is necessary to conduct research on safety, considering the various characteristics of sporting events.

## 5. Conclusions

We found that sports activity habits had a positive effect on the awareness of sports safety as well as a positive effect on the intention to complete safety education. At the same time, awareness of sports safety had a positive effect on all three pathways towards the intention to complete safety education. In addition, awareness of sports safety had a partial mediating effect between sports activity habits and the intention to complete safety education. In particular, we found that sports activity habits of adolescents were important antecedent factors that can affect their intention to complete safety education and their awareness of sports safety.

Based on these results, we believe that activity lists and guidelines regarding safety in sports should be provided. These should be divided into general and individual sports so that adolescents can develop appropriate sports activity habits. Concurrently, we need to find ways to help adolescents assess and manage themselves in ways that generate appropriate sports lifestyles. Ultimately, we need to improve sports safety awareness by paying attention to the development and implementation of sports safety education programs for adolescents as a national policy and, through this, increase their willingness to complete safety education. For this to happen, we need to take the lead in creating a safety culture and atmosphere—one where actual sports sites can function as places for sports safety education. In the future, follow-up studies should be conducted based on the safety activities and routines of elite athletes in sports situations, in order to systematically report on effective accident prevention strategies for elite athletes.

## Figures and Tables

**Table 1:** General characteristics of participants.

Variable	Classification	Number of Participants	%
Gender	Male	2895	73.7
	Female	1033	26.3
Classification of player	Professional sports player	666	17.0
	Club sports player	3262	83.0
Drinking	Have drinking experience	3803	96.8
	No drinking experience	125	3.2
Smoking	Have smoking experience	3791	95.7
	No smoking experience	167	4.3
Average sleep time	6 h or less	827	21.1
	7 h	1313	33.4
	8 h	1356	34.5
	Over 9 h	432	11.0
Frequency of sports activity per week	Everyday	1697	43.2
	4 or 5 times a week	1644	41.9
	2 or 3 times a week	338	8.6
	Less than once a week	249	6.3
Average exercise time per session	60 min or less	575	14.6
	61 to 90 min	256	6.5
	91 to 120 min	887	22.6
	121 to 150 min	357	9.1
	151 to 180 min	754	19.2
	181 to 210 min	144	3.7
	211 to 240 min	424	10.8
	Over 241 min	531	13.5
Total		3928	100.0

Tested by frequency analysis.

**Table 2:** Reliability analysis.

Latent Variable	Cronbach’s α
Sports activity habits	0.811
Sports safety awareness	0.868
Intention to complete safety education	0.685

**Table 3:** Model fit of confirmatory factor analysis.

	χ^2^/Degree of Freedom	Root Mean Square Residual	Goodness-of-Fit-Index	Incremental Fit Index	Comparative Fit Index	Root Mean Square Error of Approximation
Proposed model	60.708	0.080	0.775	0.685	0.684	0.123
Revised model	19.638	0.043	0.943	0.907	0.907	0.069

Tested by confirmatory factor analysis.

**Table 4:** Result of confirmatory factor analysis.

Latent Variable	Observation Variable		Non-SRW	SE	Critical Ratio	*p*	SRW	CR	AVE
Sports activity habits	→	(1) pre-exercise warm-up	0.754	0.029	26.096	<0.001 ***	0.549	0.995	0.932
	→	(2) checking the surrounding facilities for defects and safety before exercise	0.922	0.038	24.356	<0.001 ***	0.499		
	→	(3) performing appropriate exercise after determining the exercise level beforehand	1.043	0.034	30.816	<0.001 ***	0.720		
	→	(4) adequate intake of water before exercise	1.043	0.036	29.151	<0.001 ***	0.652		
	→	(6) wearing appropriate clothing and safety gear for the sport	1.086	0.042	25.842	<0.001 ***	0.542		
	→	(7) reporting pain and injuries to the coach	0.876	0.033	26.264	<0.001 ***	0.554		
	→	(8) checking environmental factors, such as weather disasters and signs before exercising	0.883	0.040	22.033	<0.001 ***	0.437		
	→	(10) engaging in cool-down exercises	1.000	-	-	<0.001 ***	0.553		
Sports safety awareness	→	(1) awareness of how to prevent sports accidents	1.000	-	-	<0.001 ***	0.883	0.993	0.977
	→	(2) awareness of how to cope with sports accidents	1.067	0.017	62.973	<0.001 ***	0.919		
	→	(3) awareness of CPR	0.530	0.022	24.356	<0.001 ***	0.388		
	→	(5) awareness of coping with musculoskeletal damage	0.673	0.024	28.525	<0.001 ***	0.447		
Intention to complete safety education	→	(1) necessity of sports safety education	1.000	-	-	<0.001 ***	0.536	0.990	0.972
	→	(2) intention to complete safety education	1.829	0.071	25.902	<0.001 ***	0.832		
	→	(3) intention to complete a specific safety education program	1.261	0.048	26.272	<0.001 ***	0.623		

*** *p* < 0.001, tested by confirmatory factor analysis. SRW, standardized regression weight; SE, standard error; CR, critical ratio; AVE, average variance extracted.

**Table 5:** Discriminant validity verification.

Latent Variable	Correlation			AVE
	Sports Activity Habits	Sports Safety Awareness	Intention to Complete Safety Education	
Sports activity habits	1.000	-	-	0.932
Sports safety awareness	0.504 ***	1.000	-	0.977
Intention to complete safety education	0.388 ***	0.336 ***	1.000	0.972

*** *p* < 0.001, tested by correlation analysis; AVE, average variance extracted.

**Table 6:** Descriptive statistical analysis (maximum score: 5 points).

Latent Variable	Observation Variable	Mean	SD	Skewness	Kurtosis
Sports activity habits	(1) pre-exercise warm-up	4.49	0.672	−1.348	2.272
	(2) checking the surrounding facilities for defects and safety before exercise	3.84	0.906	−0.778	0.502
	(3) performing appropriate exercise after determining the exercise level beforehand	4.08	0.710	−0.542	0.668
	(4) adequate intake of water before exercise	4.18	0.784	−0.751	0.318
	(6) wearing appropriate clothing and safety gear for the sport	4.02	0.983	−0.993	0.487
	(7) reporting pain and injuries to the coach	4.06	0.774	−0.642	0.458
	(8) checking environmental factors, such as weather disasters and signs before exercising	3.62	0.991	−0.528	−0.085
	(10) engaging in cool-down exercises	4.15	0.885	−1.222	1.638
Sports safety awareness	(1) awareness of how to prevent sports accidents	3.77	0.752	−0.315	0.142
	(2) awareness of how to cope with sports accidents	3.71	0.771	−0.214	−0.116
	(3) awareness of CPR	3.74	0.907	−0.577	0.273
	(5) awareness of coping with musculoskeletal damage	3.32	1.001	−0.119	−0.466
Intention to complete safety education	(1) necessity of sports safety education	4.09	0.758	−0.512	0.165
	(2) intention to complete safety education	3.60	0.891	−0.338	0.098
	(3) intention to complete a specific safety education program	3.19	0.822	−0.084	0.624

Tested by descriptive statistical analysis; SD, standard deviation.

**Table 7:** Model fit of path analysis.

Model	χ^2^/DF	RMR	GFI	IFI	CFI	RMSEA
Proposed model	19.638	0.043	0.943	0.907	0.907	0.069

DF, degree of freedom; RMR, root mean square residual; GFI, goodness-of-fit index; IFI, incremental fit index; CFI, comparative fit index; RMSEA, root mean square error of approximation.

**Table 8:** Results of the path analysis.

Hypothesis	Path			SRC	RC	SE	CR	*p*	Verification
H1	Sports activity habits	→	Sports safety awareness	0.602	0.817	0.031	25.949	<0.001 ***	Accept
H2	Sports activity habits	→	Intention to complete safety education	0.360	0.298	0.024	12.252	<0.001 ***	Accept
H3	Sports safety awareness	→	Intention to complete safety education	0.145	0.089	0.015	5.873	<0.001 ***	Accept

*** *p* < 0.001, tested by path analysis; SRC, standardized regression coefficient; RC, regression coefficient; SE, standard error; CR; critical ratio.

**Table 9:** Model fit of hypothesis and competition models.

	χ^2^	Degree of Freedom	Tucker Lewis Index	Comparative Fit Index	Root Mean Square Error of Approximation
Hypothesis model	1708.519	87	0.888	0.907	0.069
Competition model	1897.667	88	0.876	0.869	0.072

∆χ^2^ = 189.148.

**Table 10:** Indirect effects analysis.

Path					Estimate	Standard Error	Bias-Corrected Bootstrap	
							Lower	Upper
Sports activity habits	→	Sports safety awareness	→	Intention to complete safety education	0.177	0.018	0.144	0.212

Sampling is 1000 times; bootstrap estimates are standardized data.

## Data Availability

The data presented in this study are available upon request from the corresponding author. The data were not publicly available because of the protection of personal information.
